# Integrated metabolite profiling and transcriptome analysis reveal candidate genes involved in the biosynthesis of benzylisoquinoline alkaloids in *Corydalis solida*

**DOI:** 10.5511/plantbiotechnology.24.0205a

**Published:** 2024-09-25

**Authors:** Yasuyuki Yamada, Emi Tamagaki, Nobukazu Shitan, Fumihiko Sato

**Affiliations:** 1 Laboratory of Medicinal Cell Biology, Kobe Pharmaceutical University, Kobe, Hyogo 658-8558, Japan; 2 Graduate School of Biostudies, Kyoto University, Kyoto, Kyoto 606-8502, Japan; 3 Bioorganic Research Institute, Suntory Foundation for Life Science, Soraku-gun, Kyoto 619-0284, Japan

**Keywords:** benzylisoquinoline alkaloids, *Corydalis solida*, *O*-methyltransferase, plant specialized metabolism

## Abstract

Structurally diverse benzylisoquinoline alkaloids (BIAs) are found in specific plant families, some of which are desirable for their efficient production because of their strong biological activities. *Corydalis* plants (e.g., *Corydalis yanhusuo*) of the family Papaveraceae also produce various BIAs; thus, they have been used in traditional Chinese medicine. Because metabolic engineering and synthetic biology using microorganisms are promising technologies for the effective production of useful metabolites, elucidation of the biosynthetic pathway of each BIA is indispensable. Although several enzyme genes involved in the biosynthesis of *Corydalis* BIAs have recently been isolated, many remain unknown, such as the protoberberine alkaloid *C*-methyltransferase involved in the biosynthesis of corydaline, one of the main BIAs found in the tubers of *Corydalis* plants. In this study, we performed transcriptome analysis combined with metabolite profiling of different tissues of *Corydalis solida*. Based on the high accumulation of several BIAs, including protopine, allocryptopine, and corydaline, genes encoding putative biosynthetic enzymes, including cytochrome P450, methyltransferase, and oxidase proteins, that were highly expressed in the tubers were screened. Two OMT genes, *CsOMT1* and *CsOMT2*, were highly expressed in the tuber, and further characterization using crude enzyme preparations demonstrated that CsOMT1 showed 7-*O*-methylation activity against reticuline, whereas CsOMT2 catalyzed 9-*O*-methylation of scoulerine, followed by 2-*O*-methylation of tetrahydrocolumbamine. Our findings provide valuable information for the isolation of novel biosynthetic enzyme genes in *Corydalis* species.

## Introduction

Benzylisoquinoline alkaloids (BIAs) are structurally diverse chemicals comprising approximately 2,500 structures found in specific plant families such as Ranunculaceae, Berberidaceae, Papaveraceae, and Rutaceae (Sato 2020). Biologically active BIAs, including the analgesics morphine and codeine, antimicrobials berberine and sanguinarine, and anti-amoebic emetine, are often present at low concentrations in plants and are expensive to synthesize chemically (Hagel and Facchini 2013). Metabolic engineering and reconstruction of biosynthetic pathways in microorganisms via synthetic biology are promising technologies for the effective production of valuable compounds (Diamond and Desgagné-Penix 2016; Minami 2013; Sato 2013). Some useful BIAs including reticuline, an important intermediate of BIAs, thebaine, magnoflorine, and sanguinarine can be produced in *Escherichia coli* and *Saccharomyces cerevisiae* (Fossati et al. 2014; Galanie et al. 2015; Minami et al. 2008; Nakagawa et al. 2016). Metabolic engineering improves BIA productivity in cultured plant cells by overexpressing rate-limiting enzymes and transcription factor genes (Apuya et al. 2008; Inui et al. 2007; Yamada et al. 2017). The elucidation of biosynthetic pathways, such as berberine and magnoflorine in *Coptis japonica* (Ranunculaceae), morphine and noscapine in *Papaver somniferum* (Papaveraceae), and sanguinarine in *Eschscholzia californica* (Papaveraceae), enables us to alter the genetics and to develop an efficient production process for important BIAs (Hagel and Facchini 2013; Sato 2020; Singh et al. 2019).

*Corydalis* species in Papaveraceae produce diverse BIAs, including berberine, tetrahydropalmatine, corydaline, and some *Corydalis* plants (e.g., *Corydalis yanhusuo*, Papaveraceae), which have been used in traditional Chinese medicine because of their analgesic effects and antimicrobial/viral activities (Alhassen et al. 2021; Deng et al. 2021; Xu et al. 2021; Zielińska et al. 2020). The biosynthetic pathway of BIAs in *Corydalis* species has been recently investigated (Supplementary Figure S1). Several *O*-methyltransferases (OMTs) and (*S*)-*N*-methylcoclaurine 3′-hydroxylase involved in the conversion of (*S*)-norcoclaurine to (*S*)-reticuline and methylenedioxy bridge-forming cytochrome P450 enzymes (CYP719 family proteins) involved in the conversion of (*S*)-scoulerine to (*S*)-stylopine and (*S*)-tetrahydrocolumbamine, the intermediates of sanguinarine and berberine, respectively, have been isolated from *C. yanhusuo* (Bu et al. 2022; Liu et al. 2021, 2023). However, genes involved in the biosynthesis of several BIAs have not yet been isolated or characterized from *Corydalis* plants. The isolation of various genes encoding key enzymes involved in *Corydalis* BIAs will contribute to the efficient production of valuable BIAs using microorganisms.

In this study, we used *Corydalis solida* (Papaveraceae) to identify the genes involved in BIA biosynthesis because it is relatively easy to obtain and produces various types of BIAs (Zielińska et al. 2020). Different accumulations of metabolites and transcripts among tissues in non-model medicinal plants provide insights into the isolation of key genes involved in the biosynthesis of specialized plant metabolites (Nett et al. 2020). Our metabolite profiling and transcriptome analyses of different tissues in *C. solida* indicated that tissue-specific BIAs accumulated and the related biosynthetic enzyme genes were highly expressed in specific tissues. Further characterization indicated that the candidate OMT genes were highly expressed in the tubers and were confirmed to be involved in the production of several BIAs using recombinant enzyme assays. These findings provide valuable information for the exploration of novel BIA biosynthetic enzyme genes in *Corydalis* species.

## Materials and methods

### Plant material

*Corydalis solida* was purchased from a local gardening store and identified by comparison with the sequences of maturase K (*matK*)-like and ribulose-1,5-bisphosphate carboxylase/oxygenase large subunit (*rbcL*) genes of *C. solida* registered in NCBI (accession numbers: KP715392 and KM360733, respectively). The plant was separated into leaf blade, petiole, and tuber, and each sample was frozen in liquid nitrogen and stored at −80°C (Supplementary Figure S2).

### Chemicals

(*RS*)-Reticuline, (*RS*)-norreticuline, and (*S*)-scoulerine were provided by MITSUI CHEMICALS (Tokyo, Japan). Palmatine chloride was gifted by Dr. K. Iwasa (Kobe Pharmaceutical University). Columbamine and dehydrocorydaline were purchased from ChemFaces Biochemical (Wuhan, China) and MedChemExpress LLC (Monmouth Junction, NJ, USA), respectively. (*S*)-Laudanosoline, sanguinarine chloride, and corydaline were purchased from Sigma-Aldrich (St. Louis, MO, USA). Protopine, chelerythrine chloride, jatrorrhizine chloride, and L-tetrahydropalmatine were purchased from the Tokyo Chemical Industry (Tokyo, Japan). Epiberberine chloride was purchased from Nagara Science (Gifu, Japan). (−)-Tetrahydrocolumbamine and allocryptopine were purchased from Cayman Chemical (Ann Arbor, MI, USA). Berberine chloride, coptisine chloride, and AdoMet were purchased from FUJIFILM Wako Pure Chemical Industries, Ltd. (Osaka, Japan).

### Metabolite analysis of *C. solida* tissue extracts

Each tissue was ground in liquid nitrogen and extracted in 5 µl mg^−1^ of methanol containing 0.01 N HCl at room temperature overnight. After centrifugation at 15,000×g for 20 min and filtration using a 0.45 µm cosmospin filter H (Nacalai Tesque, Kyoto, Japan), the flowthroughs were analyzed using an ACQUITY ultra-performance liquid chromatography-mass spectrometry (UPLC-MS) system with a QDa mass detector (Waters Corp., Milford, MA, USA) under the following conditions: column, ACQUITY UPLC BEH C18 column (2.1×100 mm, 1.7 µm); H_2_O (solvent A)/acetonitrile (solvent B) gradient containing 0.1% formic acid, 0–1 min, 10–15% B; 1–12 min, 15–50% B; 12–13.5 min, 50–80% B; 13.5–15 min, 80–10% B; and 15–16 min, 10% B; UV detection, absorbance measurement from 210 nm to 400 nm with an PDA eλ Detector (Waters Corp.); flow speed, 0.3 ml min^−1^; column temperature, 40°C; injection volume, 2 µl. The QDa conditions were set as follows: positive ion scan mode=250–400 Da; cone voltage=15 V, capillary voltage=0.8 kV, and source temperature=600°C. Corydaline and allocryptopine (mass-to-charge ratio [*m*/*z*]=370), dehydrocorydaline (*m*/*z*=366), protopine (*m*/*z*=354), sanguinarine (*m*/*z*=332), berberine and epiberberine (*m*/*z*=336), coptisine (*m*/*z*=320), palmatine (*m*/*z*=352), jatrorrhizine and columbamine (*m*/*z*=338), tetrahydrocolumbamine (*m*/*z*=342), and tetrahydropalmatine (*m*/*z*=356) were detected using single-ion recording mode (positive), and chelerythrine (*m*/*z*=348) was detected using scan mode (positive). Each peak was identified based on the retention time and mass-to-charge ratio of authentic standards.

### RNA sequencing

Total RNA was extracted from *C. solida* leaf blades, petioles, and tubers using an RNeasy Plant Mini Kit (Qiagen, Hilden, Germany). RNA sequencing was performed by Hokkaido Biosystem Science (Sapporo, Japan), according to a previously described procedure. After confirming the quality and quantity of the RNA samples using an Agilent 2100 Bioanalyzer (Agilent Technologies, Santa Clara, CA, USA), a sequence library was prepared using a TruSeq Stranded mRNA Sample Prep Kit according to the standard protocol (Illumina, San Diego, CA, USA). The paired ends of the 100 bp RNA library were sequenced using an Illumina HiSeq 2500 to cover more than 4 Gb reads. After removing adapters and low-quality sequences, de novo assembly and read mapping were performed using Trinity ver. 2.0.6 and Bowtie ver. 1.1.1, respectively. Expression profiling was performed using RSEM ver. 1.2.19 and edgeR ver. 3.8.6. with gene annotation using BLAST+ ver. 2.2.29+. The Illumina sequencing data in this study can be found in the DDBJ Sequence Read Archive (DRA) under BioProject accession number PRJDB17175.

### Isolation of candidate OMT genes

Full-length TR39830 and TR11683 cDNAs were amplified by PCR using specific primers (Supplementary Table S1) and template cDNAs were synthesized from the total RNA of *C. solida* tubers using the PrimeScript RT Reagent Kit with gDNA Eraser (Takara Bio, Kusatsu, Japan). The PCR products were subcloned into a pGEM-T Easy vector (Promega, Madison, WI, USA), and expression vectors were constructed in pET-22b (+) (Merck Millipore, Burlington, MA, USA) using an In-Fusion HD Cloning Kit (Takara Bio). A phylogenetic tree was created using the neighbor-joining method, Jones–Thornton–Taylor model, and 1,000 bootstrap replications using MEGA 11 software (https://www.megasoftware.net/, Tamura et al. 2021). Sequence data can be found in the GenBank/DDBJ data libraries under the accession numbers LC790672 (CsOMT1) and LC790673 (CsOMT2).

### Heterologous expression of OMTs in *Escherichia coli*

BL21 (DE3) *E. coli* harboring the expression vectors were grown in Luria Bertani (LB) medium at 37°C and 200 rpm. After the addition of 1 mM isopropyl β-D-thiogalactopyranoside at OD_600_=0.7–0.8, the *E. coli* cells were further grown at 16°C for 24 h. Recombinant proteins were extracted from the harvested *E. coli* cells by sonication in extraction buffer [100 mM Tris-HCl (pH 7.5), 10% glycerol, 1 mM dithiothreitol]. Homogenates were centrifuged at 16,000×g, for 20 min at 4°C and the supernatants were used as crude enzymes for the enzyme assay and SDS-PAGE analysis.

### In vitro enzyme assay

Catalytic activity of OMTs was measured in 50 µl of 100 mM Tris-HCl (pH 7.5), 10% glycerol, 1 mM AdoMet, 25 mM sodium ascorbate, 1 mM dithiothreitol, 100 µM substrate, and 30 µg of crude protein at 30°C for 18 h. The enzymatic reactions were stopped by the addition of 50 µl methanol. After the centrifugation at 15,000×g for 5 min and filtration with a 0.45 µm cosmospin filter (Nacalai Tesque), the flowthrough was analyzed using the UPLC-MS with the ACQUITY UPLC BEH C18 column (2.1×100 mm, 1.7 µm). When the reaction products of scoulerine, norreticuline, and reticuline were analyzed, the UPLC conditions were set as follows: H_2_O (solvent A)/acetonitrile (solvent B) gradient containing 0.01% acetate, 0–9 min, 5–40% B; 9–12 min, 40–50% B; and 12–15 min, 50–5% B; flow rate, 0.3 ml min^−1^; injection, 2 µl; and column temperature, 40°C. The UPLC conditions for the analysis of norlaudanosoline and laudanosoline reaction products were set as follows: H_2_O (solvent A)/acetonitrile (solvent B) gradient containing 0.01% acetate, 0–4 min, 2–6% B; 4–14 min, 6–35% B; 14–15 min, 35–98% B; 15–18 min, 98–2% B; and 18–20 min, 2% B; flow rate, 0.3 ml min^−1^; injection, 5 µl; and column temperature, 30°C. The QDa conditions were set as follows: cone voltage=15–50 V, capillary voltage=0.8 kV, and source temperature=600°C.

## Results

### BIA profile in *C. solida*

**Figure 1:**
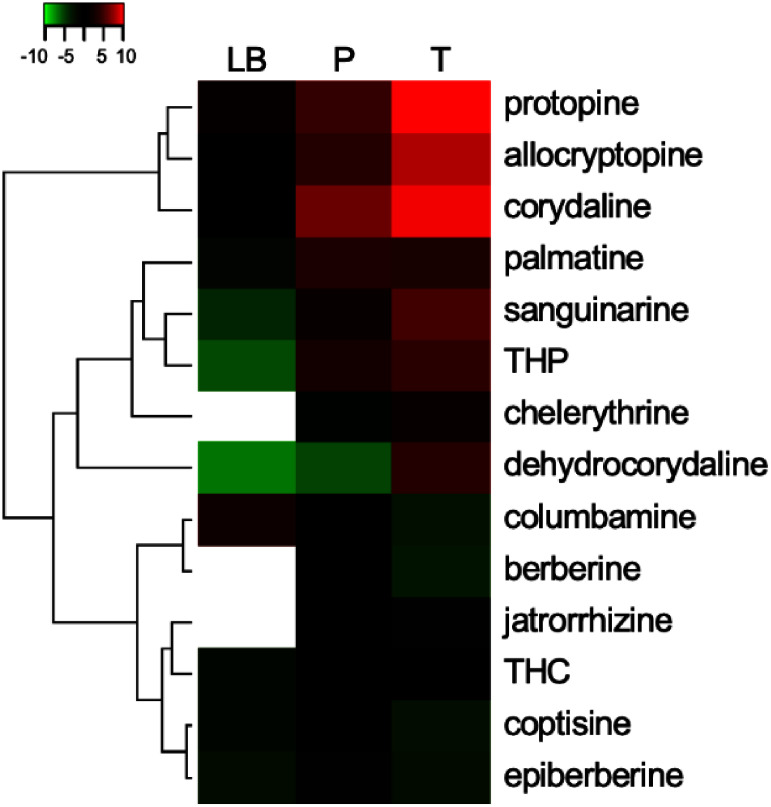
Figure 1. Heatmap diagram of benzylisoquinoline alkaloid profiles in *Corydalis solida* tissues. The relative abundance of each benzylisoquinoline alkaloid (BIA) was determined by ultra-performance liquid chromatography-mass spectrometry (UPLC-MS) and normalized to the corydaline content (µg g^−1^ fresh weight) in the leaf blade as Log2 fold change value. LB, leaf blade; P, petiole; T, tuber. The white box shows BIAs that were not detected.

To compare the metabolite content among *C. solida* tissues (leaf blade, petiole, and tuber), the methanol extracts were analyzed using UPLC-MS. The accumulation of the 14 BIAs in each tissue sample was quantitatively calculated using a standard curve of authentic standards. As shown in Figure 1, more protopine, allocryptopine, corydaline, dehydrocorydaline, sanguinarine, and chelerythrine accumulated in the tubers of *C. solida* than in the petioles and leaf blades. Protopine was the most abundant analyte (339.9 µg g^−1^ fresh weight (FW)), followed by corydaline (245.6 µg g^−1^ FW) and allocryptopine (95.7 µg g^−1^ FW). These three compounds accounted for over 90% of the total tuber BIAs. Metabolite analysis further revealed differences in tetrahydroprotoberberine- and protoberberine-type BIA profiles among the three tissues; tetrahydropalmatine (THP) and tetrahydrocolumbamine (THC) showed high accumulation in the tuber, whereas palmatine and columbamine showed high accumulation in the petiole and leaf blade, respectively. The content of other BIAs containing berberine, epiberberine, coptisine, and jatrorrhizine were higher in petiole than that in leaf blade and tuber, though the abundance was quite low (<0.6 µg g^−1^ FW) compared with corydaline (34.4 µg g^−1^ FW), protopine (8.6 µg g^−1^ FW), and allocryptopine (5.5 µg g^−1^ FW) in petiole.

### Exploration of candidate genes encoding biosynthetic enzymes in BIA biosynthesis

To examine the expression profiles of genes in different tissues of *C. solida*, transcriptome analysis was performed using RNA samples prepared from leaf blades, petioles, and tubers. Approximately 45 million paired-end reads with more than 96% Q30 bases were obtained by RNA sequencing and 130,751 contigs with a total of 110,606,782 bp, an average length of 846 bp, and an N50 length of 1,411 bp were generated by de novo assembly.

We compared transcript abundance among different tissues using transcripts per million (TPM) values to screen for candidate genes involved in the biosynthesis of BIAs that accumulate in specific tissues of *C. solida* (Supplementary Figure S3). A total of 7,709 contigs with a threshold of TPM in tubers >20 were first extracted, and then 439 contigs with log2 fold change (FC) values >1 in tubers versus petioles, followed by petioles versus leaf blades, and *p* value <0.05. A total of 370 genes were found in contigs that were excluded because of a lack of annotated protein genes. These 370 genes were highly correlated with tissue expression and BIA accumulation (tuber>petiole>leaf blade). The screened contigs contained genes encoding enzymes such as cytochrome P450s, methyltransferases, and reductases, which are putatively involved in BIA biosynthesis (representative genes are listed in Table 1) (Supplementary Figure S1). For example, TR58023, TR11143, TR28144, and TR61556 contig genes were annotated as (*S*)-cheilanthifoline synthase (*CHS*), (*S*)-stylopine synthase (*STS*), (*S*)-tetrahydroprotoberberine *N*-methyltransferase (*TNMT*), and (*S*)-*N*-methylstylopine 14-hydroxylase (*MSH*), respectively, and were highly expressed in tuber with >570 TPM values. Because CHS, STS, TNMT, and MSH are essential enzymes for the biosynthesis of protopine and allocryptopine (Sato 2020), the high TPM values of the four genes were correlated with the high accumulation of these BIAs in the tuber. TR11683 encodes an enzyme annotated as (*S*)-scoulerine 9-*O*-methyltransferase (SOMT), which probably plays a crucial role in the biosynthesis of corydaline from (*S*)-scoulerine and exhibited a high TPM value (>900) in the tuber. Although guessing the specific function is difficult based on annotation, TR39830- and TR26557-encoding enzymes may play an important role in the biosynthesis of BIAs that are mainly found in the tuber because of their high TPM values (>1,100).

**Table 1:** Table 1. Representative candidate genes involved in BIA biosynthesis.

Gene ID	TPM (LB)	TPM (P)	TPM (T)	Annotation
TR39830	4.98	534.44	1209.5	Catechol *O*-methyltransferase [*Thalictrum tuberosum*]
TR26557	1.28	19.84	1152.07	Tropinone reductase At1g07440, partial [*Anthurium amnicola*]
TR11683	0.1	68.53	922.01	(*S*)-scoulerine 9-*O*-methyltransferase [*Thalictrum flavum* ssp. glaucum]
TR58023	6.72	303.73	832.87	Cheilanthifoline synthase; Short=CHS
TR11143	11.11	266.68	633.98	(*S*)-stylopine synthase 1; Short=STS [*Eschscholzia californica*]
TR61556	0	32.45	583.58	Methyltetrahydroprotoberberine 14-monooxygenase
TR27890	0.66	100.75	519.09	Coclaurine *N*-methyltransferase [*Coptis japonica*]
TR28144	0	57.59	489.06	(*S*)-tetrahydroprotoberberine *N*-methyltransferase; Short=EcTNMT
TR30962	1.47	231.31	479.59	3′-hydroxy-*N*-methyl-(*S)*-coclaurine 4′-*O*-methyltransferase 2; Short=4′-OMT2
TR45255	2.03	177.04	448.73	(*S*)-norcoclaurine synthase, partial [*Corydalis saxicola*]
TR28583	3.31	94.62	413.11	Putative (*S*)-*N*-methylcoclaurine 3′-hydroxylase, partial [*Papaver bracteatum*]
TR8297	0.1	11.22	343.31	Coclaurine *N*-methyltransferase [*Coptis japonica*]
TR9833	1.4	52.85	293.81	*S*-adenosyl-L-methionine:*O*-methyltransferase [*Papaver somniferum*]
TR21866	0.91	81.17	259.27	Cytochrome p450 79a2 [*Theobroma cacao*]
TR17522	0.47	3.7	222.97	Cytochrome P450 71A1-like [*Asparagus officinalis*]
TR15306	0.16	8.84	115.09	Aldo/keto reductase [*Corchorus olitorius*]
TR25006	0.04	15.52	89.82	FAD-dependent oxidoreductase, partial [*Papaver somniferum*]
TR29432	0.04	1.66	74.63	Cytochrome P450 71A1 [*Morus notabilis*]
TR8311	0	0.73	56.63	Cytochrome P450, partial [*Coptis japonica* var. dissecta]
TR14053	2.66	22.59	56.21	FAD-dependent oxidoreductase [*Papaver somniferum*]

LB, leaf blade; P, petiole; T, tuber.

### Isolation and phylogenetic analysis of CsOMTs

**Figure 2:**
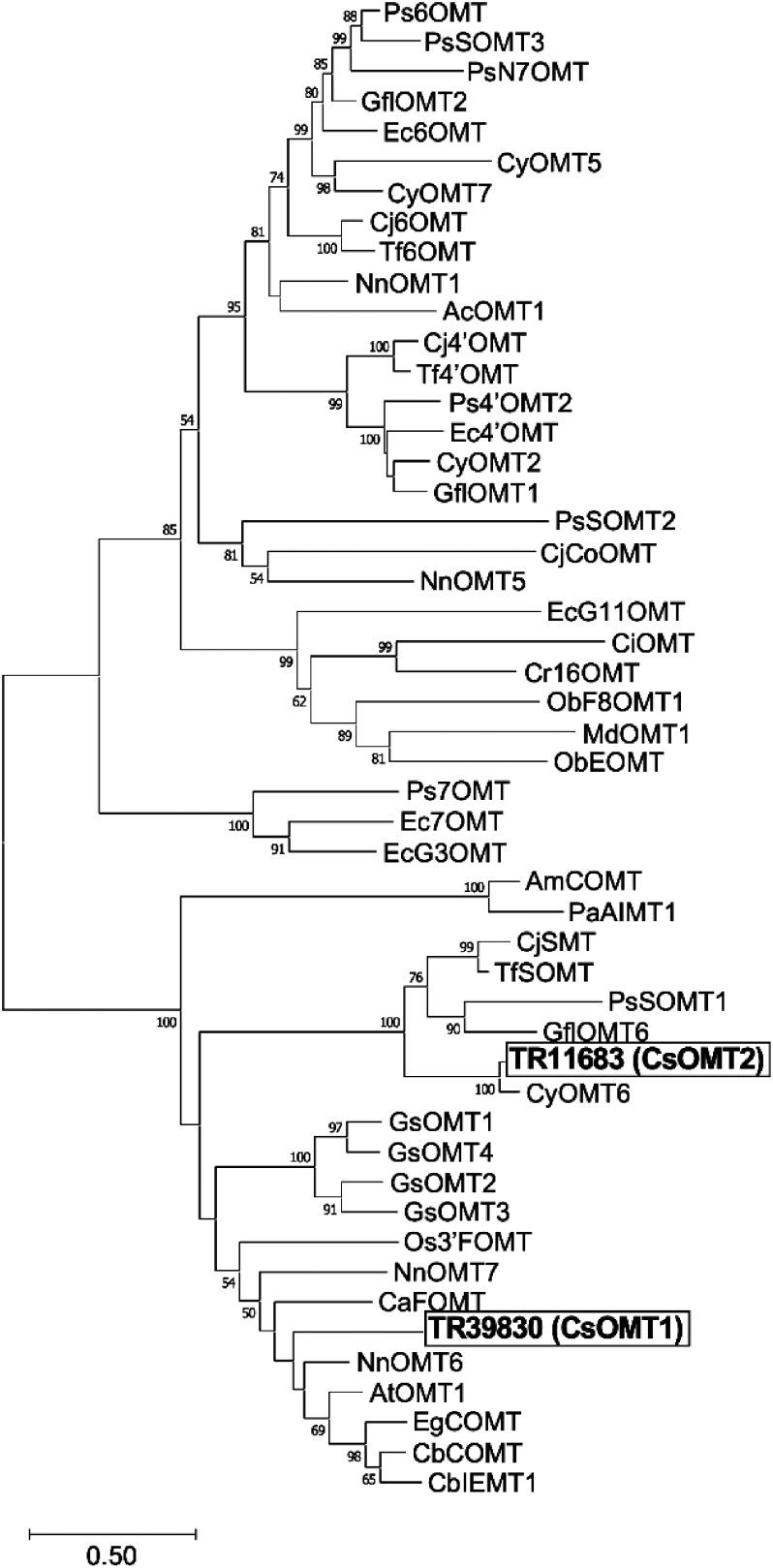
Figure 2. Phylogenetic relationships of CsOMT1 and CsOMT2 with other functionally characterized OMTs from different plant species. An NJ phylogenetic tree was constructed from the full amino acid sequences of *O*-methyltransferases (OMTs) using MEGA11 software. Bootstrap confidence values from 1,000 replicates are indicated on the branches. The scale bar shows 0.2 amino acid substitutions per site. Accession numbers for the OMT sequences are listed in Supplementary Table S2.

**Figure 3:**
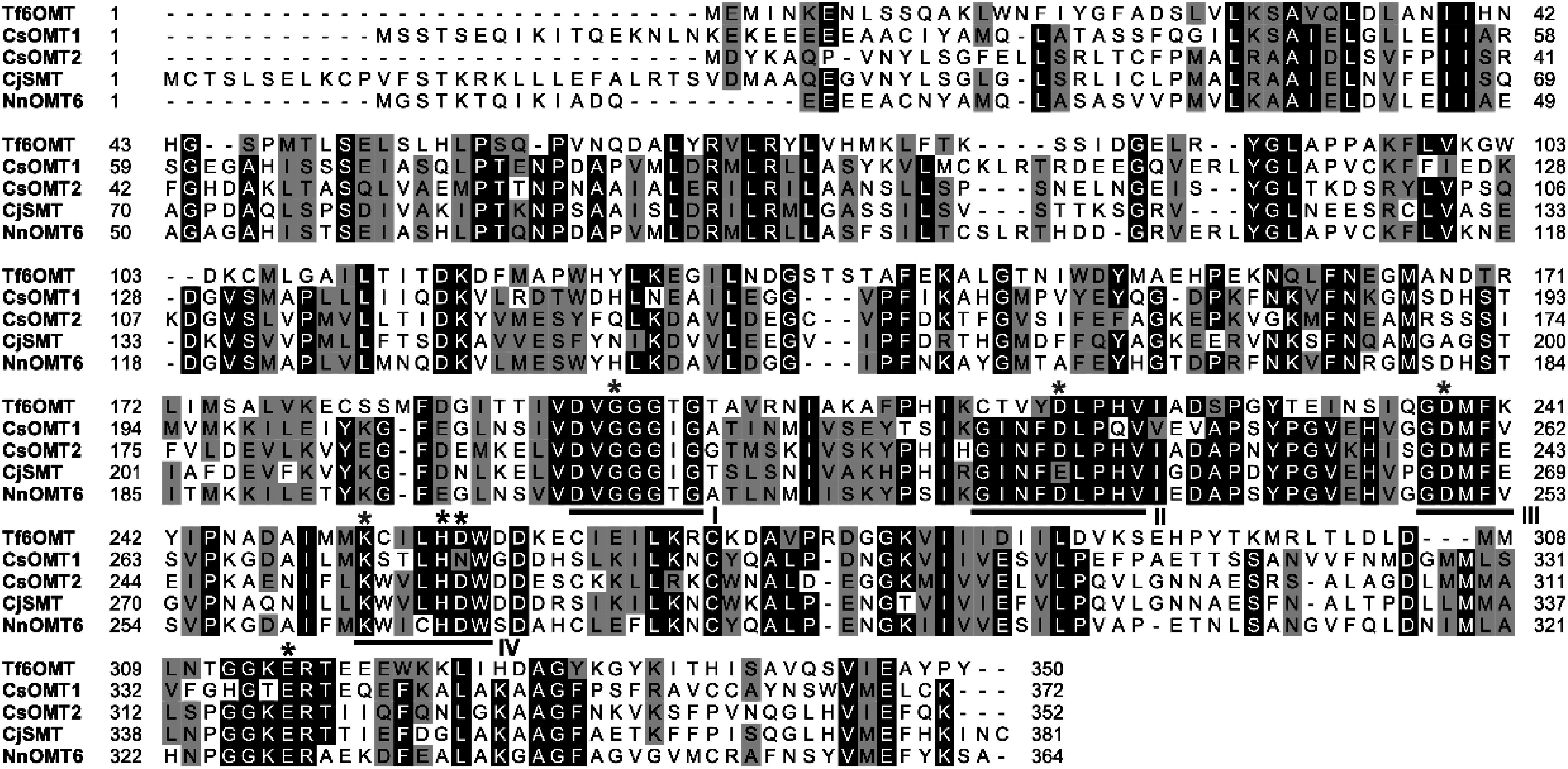
Figure 3. Amino acid sequence alignment of CsOMT1 and CsOMT2 with other OMTs. The sequences were aligned using BioEdit software. Amino acids shaded in black are identical, whereas those shaded in gray are similar. Black and gray asterisks indicate key residues related to catalytic (His^256^, Asp^257^, and Glu^315^) and *S*-adenosylmethionine (SAM) binding (Gly^195^, Asp^218^, Asp^238^, and Lys^252^), as reported for *T. flavum* 6OMT, respectively. Conserved motifs I–IV, which are involved in SAM binding, are underlined.

To demonstrate that the candidate enzymes truly have catalytic activity on BIA substrates, we isolated the full-length cDNAs of TR39830 and TR11683, designated *CsOMT1* and *CsOMT2*, respectively, and characterized their enzymatic functions. Phylogenetic analysis revealed that the amino acid sequences of CsOMT1 and CsOMT2 were similar to those of known OMTs (Figure 2). CsOMT1 showed high similarity to *Clarkia breweri* caffeic acid 3-*O*-methyltransferase (CbCOMT; 61% identity) and *Nelumbo nucifera* *O*-methyltransferase 6 (NnOMT6; 66% identity), whereas CsOMT2 showed high similarity to *Thalictrum flavum* and *C. japonica* scoulerine 9-*O*-methyltransferases (TfSOMT and CjSMT; 62% identity), and *Glaucium flavum* *O*-methyltransferase 6 (GflOMT6; 58% identity). Multiple sequence alignments of the functionally characterized Tf6OMT, CjSMT, NnOMT6, and CsOMTs showed high conservation of the key amino acid residues (His^256^, Asp^257^, and Glu^315^) in the catalytic domain, except for the D257N substitution in CsOMT1 (Figure 3). CsOMT1 and CsOMT2 contain four conserved motifs (I–IV), including the key residues (Gly^195^, Asp^218^, Asp^238^, and Lys^252^) involved in *S*-adenosylhomocysteine/*S*-adenosylmethionine (SAH/SAM) binding (Morris and Facchini 2019).

### Enzymatic activity of CsOMT1 and CsOMT2

To examine the enzymatic properties of CsOMT1 and CsOMT2, recombinant proteins were produced in *E. coli* using the pET expression system, and an enzyme assay was performed using the soluble fraction of *E. coli* crude extracts, including CsOMT1 and CsOMT2, which displayed a molecular mass of approximately 41 kDa (Supplementary Figure S4). The structures of the five BIA substrates (norlaudanosoline, laudanosoline, reticuline, norreticuline, and scoulerine) used in the enzymatic assays are shown in Supplementary Figure S5. Both CsOMT1 and CsOMT2 catalyze *O*-methylation of reticuline. Furthermore, CsOMT1 weakly catalyzes the *O*-methylation of laudanosoline, whereas CsOMT2 catalyzes the *O*-methylation of scoulerine and norreticuline.

**Figure 4:**
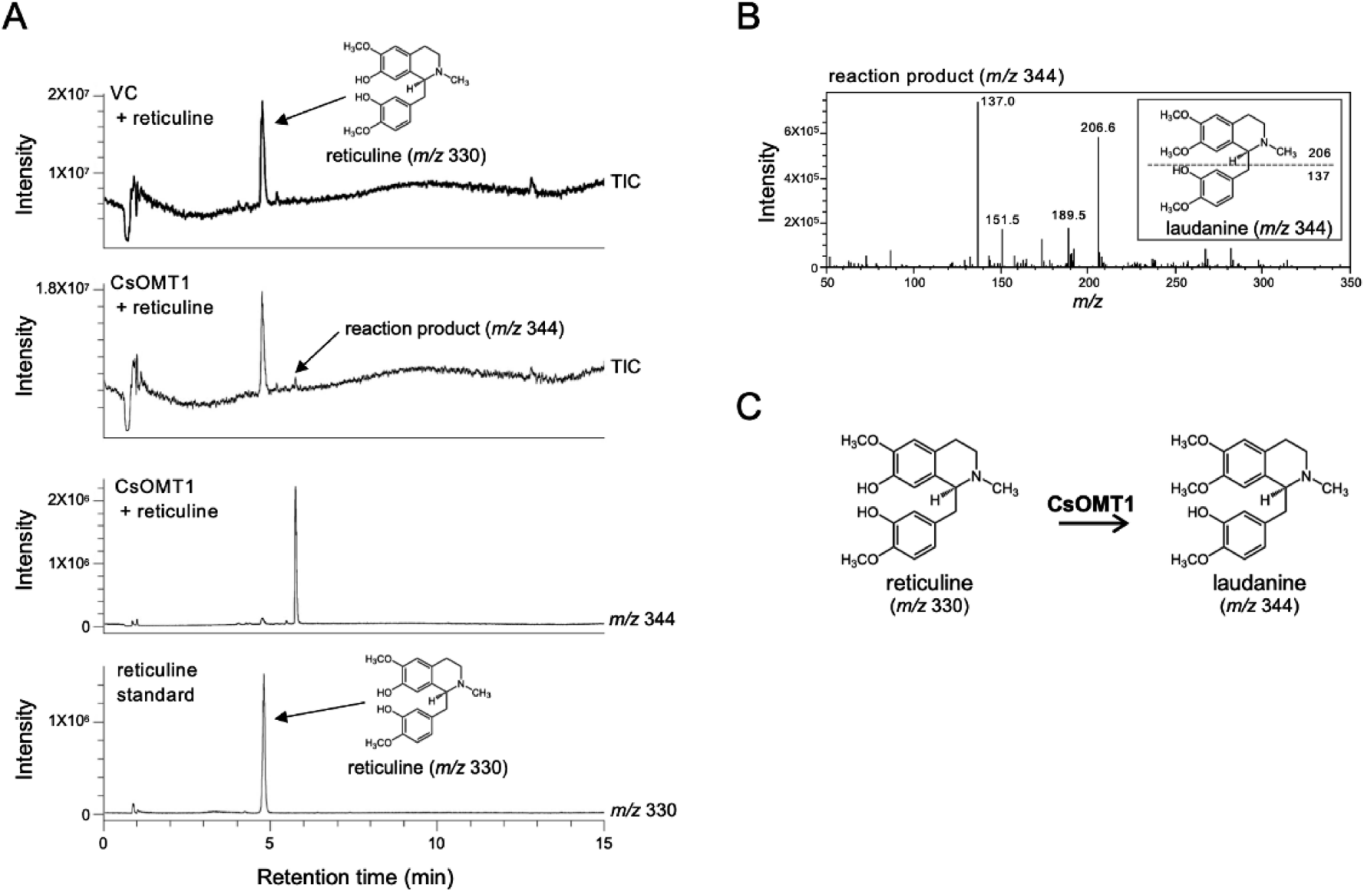
Figure 4. Enzymatic activity of CsOMT1 crude protein. (A) UPLC-MS chromatograms of the crude enzyme reactions of CsOMT1 with reticuline as a substrate. Total ion chromatogram (TIC) and single ion recording (SIR) at *m*/*z* 344 and *m*/*z* 330 were presented. (B) MS fragment pattern of reaction product of CsOMT1 with reticuline. (C) CsOMT1 catalyzes the 7-*O*-methylation of reticuline (*m*/*z* 330) to laudanine (*m*/*z* 344).

When the crude CsOMT1 protein was reacted with reticuline (*m*/*z* 330), a reaction product consistent with *O*-methylation [gain of 14 *m*/*z* with respect to the substrate] was detected (Figure 4A). The MS fragmentation pattern of the reaction product showed two major fragment peaks corresponding to the isoquinoline moiety at *m*/*z* 206 and the benzyl moiety at *m*/*z* 137 (Figure 4B), suggesting that the hydroxy group of reticuline at position 7 was *O*-methylated and that laudanine (*m*/*z* 344) was produced by CsOMT1 (Figure 4C). The reaction of crude CsOMT1 protein with laudanosoline (*m*/*z* 302) also yielded new product peaks at *m*/*z* 316; however, these products could not be identified because of their low intensity (Supplementary Figure S6).

**Figure 5:**
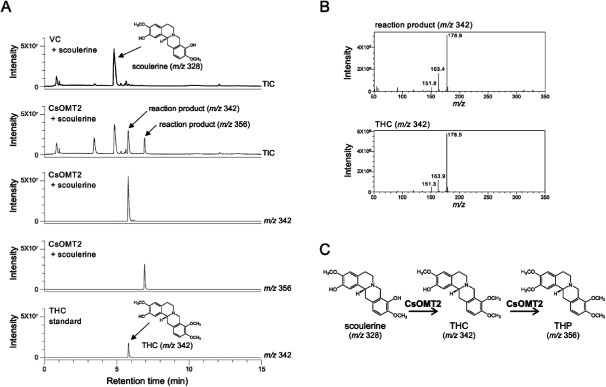
Figure 5. Enzymatic activity of CsOMT2 crude protein. (A) UPLC-MS chromatograms of the crude enzyme reactions of CsOMT2 with scoulerine. TIC and SIR at *m*/*z* 342 and *m*/*z* 356 were presented. (B) MS fragment pattern of tetrahydrocolumbamine (THC) and reaction product of CsOMT2 with scoulerine. (C) CsOMT2 catalyzes the 9-*O*-methylation of scoulerine (*m*/*z* 328) to THC (*m*/*z* 342), and subsequently catalyzes the 2-*O*-methylation of THC to tetrahydropalmatine (THP) (*m*/*z* 356).

When the crude CsOMT2 protein was reacted with scoulerine (*m*/*z* 328), two new peaks at *m*/*z* 342 and *m*/*z* 356 were detected, suggesting that single and double *O*-methylation occurred, respectively (Figure 5A). The product with an *m*/*z* of 342 was identified as THC, with a retention time of 5.8 min and an MS fragmentation pattern comparable to that of the authentic THC standard (Figure 5B). Because the hydroxyl group of THC (*m*/*z* 342) remained at position 2, the product with *m*/*z* 356 was determined to be THP. These results suggest that CsOMT2 catalyzes the 9-*O*-methylation of scoulerine, followed by the 2-*O*-methylation of THC (Figure 5C). The crude enzyme assay of CsOMT2 using norreticuline (*m*/*z* 316) showed two reaction products corresponding to *m*/*z* 330 and *m*/*z* 344 (Supplementary Figure S7). The product of the reaction between CsOMT2 and reticuline (*m*/*z* 330) exhibited two peaks, at *m*/*z* 344. One peak at 5.7 min was identified as laudanine because its retention time coincided with that of the CsOMT1 reaction product peak with reticuline, whereas the other peak was predicted to be codamine (Supplementary Figure S8). Based on the result of CsOMT2 reaction using scoulerine, it is thought that the hydroxy group of reticuline and norreticuline at position 7 and/or 3′ might be *O*-methylated. However, no reaction product could be identified by MS fragmentation patterns, owing to their low intensity.

## Discussion

Transcriptome analysis combined with metabolite profiling is a powerful method to identify candidate genes that encode biosynthetic enzymes or other proteins involved in the biosynthesis of specialized metabolites. In this study, we analyzed the tissue-specific accumulation of BIAs in *C. solida* and performed RNA sequencing and de novo assembly, followed by differential gene expression analysis, to screen candidate genes involved in the biosynthesis of BIAs mainly found in the tubers of *C. solida*. Two candidate genes encoding *O*-methyltransferases, CsOMT1 and CsOMT2, showed over 900 TPM values in the tuber, and both OMT proteins showed enzymatic activity against BIA substrates. CsOMT1 catalyzes the methylation of the C7-hydroxyl group of reticuline, whereas CsOMT2 catalyzes the methylation of the C9-hydroxyl group of scoulerine, followed by methylation of the C2-hydroxyl group of THC.

Because of their high medicinal value, the alkaloid constituents of *Corydalis* plants have been intensively investigated. Alkaloids are the most pharmacologically active compounds, with approximately 380 alkaloids have been identified in various *Corydalis* species (Deng et al. 2021). Our quantitative analysis showed that protopine was the most abundant alkaloid in the tubers of *C. solida*, which agrees with the results of a previous study (Zielińska et al. 2020). In contrast, Zielińska et al. reported that protopine accumulated the most in the aerial parts of *C. solida*, whereas the current study showed that corydaline and columbamine were the most abundant alkaloids in petioles and leaf blades, respectively. Differences in growth conditions and developmental stages may affect the alkaloid composition in the aerial parts of *C. solida*. Protopine is biosynthesized from (*S*)-reticuline, an important intermediate of BIAs, by the sequential reactions of the berberine bridge enzymes CHS, STS, TNMT, and MSH. Our transcriptome data demonstrated that the biosynthesis genes annotated as key enzymes were highly expressed in the tuber.

Metabolite analysis revealed a predominant accumulation of BIAs in *C. solida* tubers. Generally, plants compartmentalize bioactive alkaloids in vacuoles after biosynthesis to avoid toxicity. Our transcriptome data may contribute to the identification of novel genes involved in BIA transport through the co-expression analysis of biosynthetic enzyme genes. Our screening revealed 370 contigs with high tuber TPM values, including one gene that encodes a multidrug and toxic compound extrusion (MATE) transporter protein. In *C. japonica*, berberine accumulates in the vacuoles, and a MATE transporter, CjMATE1, transports berberine from the cytosol to the vacuoles (Takanashi et al. 2017).

Consistent with the phylogenetic analysis, CsOMT2 showed a catalytic function in the sequential *O*-methylation of (*S*)-scoulerine, similar to that of PsSOMT1 from the opium poppy, GflOMT6 from the yellow-horned poppy, and CyOMT6 from *C. yanhusuo* (Bu et al. 2022; Chang et al. 2015; Dang and Facchini 2012). In contrast, *Eschscholzia californica* SOMT (G3OMT), which belongs to the clade of reticuline 7-*O*-methyltransferases, showed a catalytic function for the simultaneous *O*-methylation at positions 2 and 9 of scoulerine (Purwanto et al. 2017). These results suggest *Papaver*, *Glaucium*, and *Corydalis* SOMTs obtained their catalytic activity for scoulerine 9-*O*-methylation from a common ancestral OMT, whereas *E. californica* SOMT obtained their activity independently during evolution. The enzyme assay also showed that CsOMT2 catalyzed the methylation of norreticuline (*m*/*z* 316), resulting in the detection of two new peaks at *m*/*z* 330 and *m*/*z* 344 (Supplementary Figure S7), and reticuline (*m*/*z* 330), resulting in two new peaks predicted to be laudanine (*m*/*z* 344) and codamine (*m*/*z* 344) (Supplementary Figure S8). The reaction of PsSOMT1 with norreticuline and reticuline produces norcodamine (*m*/*z* 330), tetrahydropapaverine (*m*/*z* 344), and codamine and laudanosine (*m*/*z* 358) (Dang and Facchini 2012). GflOMT6 converts norreticuline to norcodamine, norlaudanine (*m*/*z* 330), and tetrahydropapaverine, and converts reticuline to codamine, laudanine, and laudanosine, respectively (Chang et al. 2015). Based on the functions of PsSOMT1 and GflOMT6, the products of the reaction of CsOMT2 with norreticuline may be norcodamine, norlaudanine, or tetrahydropapaverine (Supplementary Figure S7). CsOMT2 did not produce the double-methylated product reticuline, suggesting that the three SOMTs have different catalytic activities against norreticuline and reticuline.

Although our phylogenetic analysis revealed that CsOMT1 belongs to a clade different from the reticuline 7-*O*-methyltransferases, CsOMT1 catalyzes the conversion of reticuline to laudanine (*m*/*z* 344). Laudanine and laudanosine (3′-*O*-methylated laudanine) were not detected in our metabolite analysis, whereas the expression level of *CsOMT1* was high. *Thalictrum tuberosum* and opium poppy OMT form heterodimers, which affect substrate specificity and catalytic activity (Frick and Kutchan 1999; Park et al. 2018). CsOMT1 may also form heterodimers with other OMTs in *C. solida* plants and may be involved in the biosynthesis of other BIAs. Sacred lotus NnOMT6, which shows high homology to CsOMT1, catalyzes the 7-*O*-methylation of simple BIAs such as coclaurine (*m*/*z* 286), 6-*O*-methylation of norcoclaurine (*m*/*z* 272), and 6-*O*-methylation of aporphine-type BIAs such as asimilobine (*m*/*z* 268) (Yu et al. 2023). The reaction products of CsOMT1 with laudanosoline may be the 6- and 7-*O*-methylated products (Supplementary Figure S6). Further biochemical characterization using purified CsOMT1, along with other candidate OMT recombinant proteins and various BIA substrates, will contribute to the elucidation of a more detailed function of CsOMT1 and the importance of homo- or heterodimers of OMT proteins. Genetic manipulation, such as the suppression of *CsOMT1* in plants, is also required to unveil the involvement of CsOMT1 in the biosynthesis of specific BIA.

Corydaline, one of the main BIAs found in the tubers of *C. solida*, is produced by the 13C-methylation of palmatine (Supplementary Figure S1). 13-Methylberberine showed stronger antiadipogenic effects than berberine in mouse 3T3-L1 cells, suggesting that 13C-methylated protoberberine alkaloids may be potential drugs for the treatment of metabolic syndromes (Chow et al. 2016). Although protoberberine alkaloid 13-*C*-methyltransferase might be cytosolic and NADPH-dependent (Rueffer et al. 1994), this key enzyme has not yet been isolated or characterized. Our integrated omics data will help us explore the key enzymes involved in the 13C-methylation of protoberberine alkaloids and achieve efficient production of potentially valuable C-methylated protoberberine derivatives using microorganisms.

## Abbreviations

BIA: benzylisoquinoline alkaloid
Cs: *Corydalis solida*
FW: fresh weight
MS: mass spectrometry
*m*/*z*: mass-to-charge ratio
OMT: *O*-methyltransferase
SAM: *S*-adenosylmethionine
THC: tetrahydrocolumbamine
THP: tetrahydropalmatine
TPM: transcript per million
UPLC: ultra-performance liquid chromatography

## Description of Supplementary Files

Supplementary Figure S1. Predicted biosynthetic pathway of BIAs in *Corydalis solida*

Supplementary Figure S2. *Corydalis solida* plant used for metabolite profiling and transcriptome analyses

Supplementary Figure S3. Number of highly expressed contigs in tuber (TPM value >20) and differentially expressed contigs (tuber>petiole>leaf blade)

Supplementary Figure S4. Coomassie Brilliant Blue-stained crude proteins in CsOMT1 (A) and CsOMT2 (B) crude extracts

Supplementary Figure S5. Chemical structure of BIA substrates used for enzyme assay

Supplementary Figure S6. Reaction products of CsOMT1 with laudanosoline

Supplementary Figure S7. Reaction products of CsOMT2 with norreticuline

Supplementary Figure S8. Reaction products of CsOMT2 with reticuline

Supplementary Table S1. Primer sequences used for isolation of cDNAs

Supplementary Table S2. Accession numbers of OMTs used for the phylogenetic analysis
