# Supplementary material for: Integrated metabolite profiling and transcriptome analysis reveal candidate genes involved in the biosynthesis of benzylisoquinoline alkaloids in Corydalis solida
Source: Plant Biotechnol (Tokyo). 2024 Sep 25;41(3):267–76. doi: 10.5511/plantbiotechnology.24.0205a (PMC11921132; doi:10.5511/plantbiotechnology.24.0205a)
Supplement: Supplementary Data [file plantbiotechnology-41-3-24.0205a-s001.pdf]

Supplementary Information

Integrated metabolite profiling and transcriptome analysis reveal candidate genes involved in the biosynthesis of benzylisoquinoline alkaloids in *Corydalis solida*

Yasuyuki Yamada[1,#,*], Emi Tamagaki[1,#,†], Nobukazu Shitan[1], Fumihiko Sato[2,3,*]

[1]Laboratory of Medicinal Cell Biology, Kobe Pharmaceutical University, Kobe, Hyogo 658-8558, Japan; [2]Graduate School of Biostudies, Kyoto University, Kyoto, Kyoto 606-8502, Japan; [3]Bioorganic Research Institute, Suntory Foundation for Life Science, Soraku-gun, Kyoto 619-0284, Japan

***Corresponding authors:**

Yasuyuki Yamada;

E-mail: yyamada@kobepharma-u.ac.jp

Fumihiko Sato;

E-mail: fsato@lif.kyoto-u.ac.jp

[#]These authors equally contributed to this work.

[†]Present address:

Graduate School of Science and Technology, Nara Institute of Science and Technology, Ikoma, Nara 630-0192, Japan

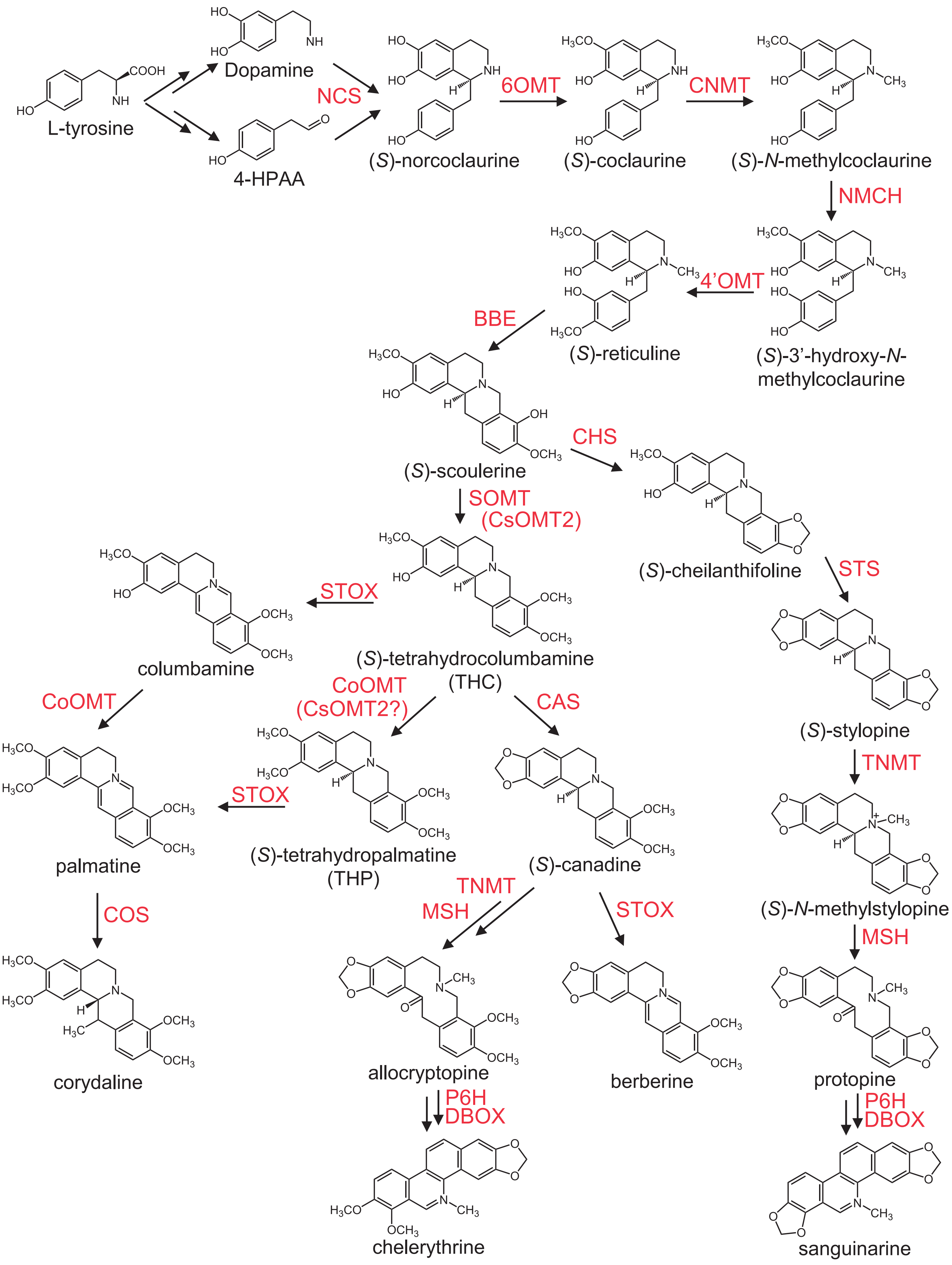

Supplementary Figure S1. Predicted biosynthetic pathway of BIAs in *Corydalis solida*.

NCS, (*S*)-norcoclaurine synthase; 6OMT, (*S*)-norcoclaurine 6-*O*-methyltransferase; CNMT, (*S*)-coclaurine-*N*-methyltransferase; NMCH, (*S*)-*N*-methylcoclaurine 3'-hydroxylase; 4'OMT, (*S*)-3'-hydroxy-*N*-methylcoclaurine 4'-*O*-methyltransferase; BBE, berberine bridge enzyme; SOMT, (*S*)-scoulerine 9-*O*-methyltransferase; CoOMT, columbamine *O*-methyltransferase; CHS, (*S*)-cheilanthifoline synthase; STS, (*S*)-stylopine synthase; CAS, (*S*)-canadine synthase; TNMT, (*S*)-tetrahydroprotoberberine *N*-methyltransferase; MSH, (*S*)-*N*-methylstylopine 14-hydroxylase; P6H, protopine 6-hydroxylase; DBOX, dihydrobenzophenanthridine alkaloid oxidase; STOX, (*S*)-tetrahydroprotoberberine oxidase; COS, corydaline synthase.

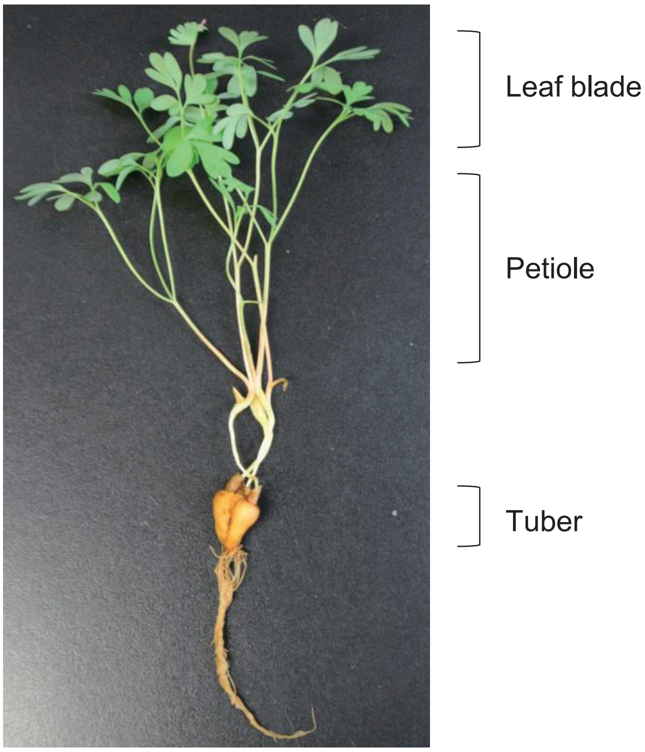

Supplementary Figure S2. *Corydalis solida* plant used for metabolite profiling and transcriptome analyses.

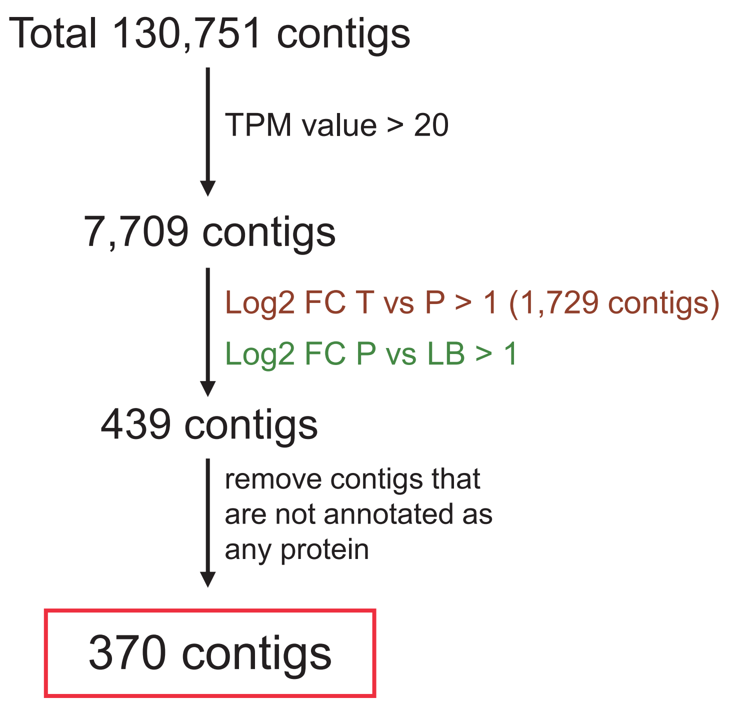

Supplementary Figure S3. Number of highly expressed contigs in tuber (TPM value > 20) and differentially expressed contigs (tuber > petiole > leaf blade). Screened 370 contig genes showed high expression in the tuber of *C. solida*. LB, leaf blade; P, petiole; T, tuber

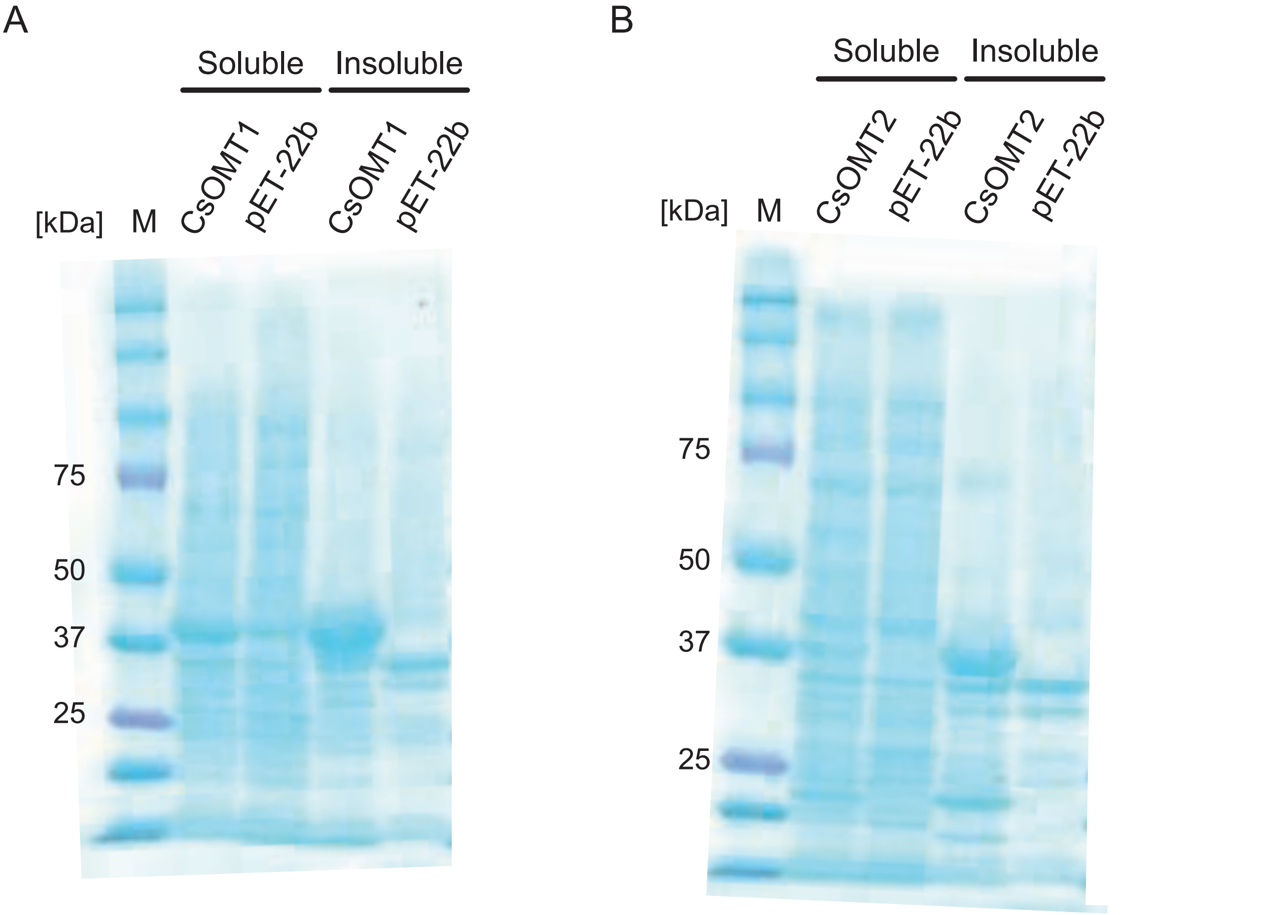

Supplementary Figure S4. Coomassie Brilliant Blue-stained crude proteins in CsOMT1 (A) and CsOMT2 (B) crude extracts.

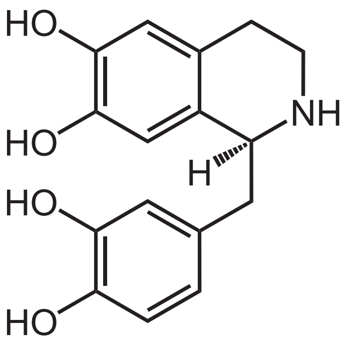

norlaudanosoline

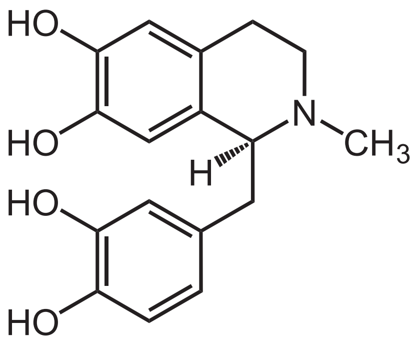

laudanosoline

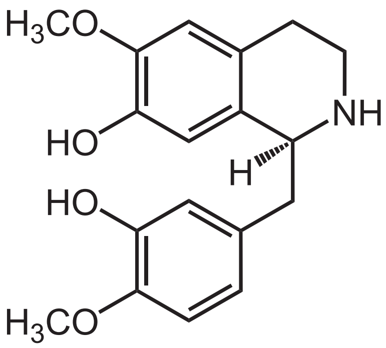

norreticuline

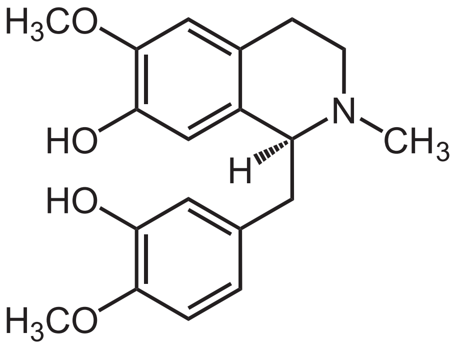

reticuline

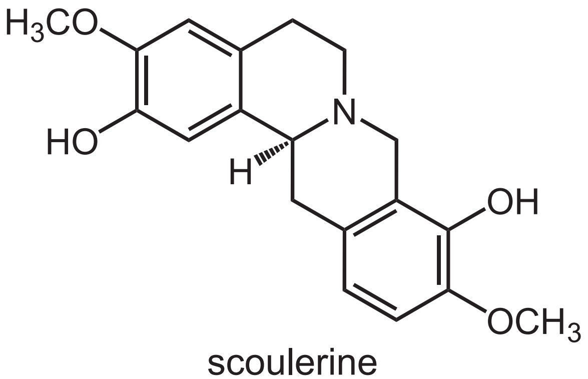

scoulerine

Supplementary Figure S5. Chemical structure of BIA substrates used for enzyme assay.

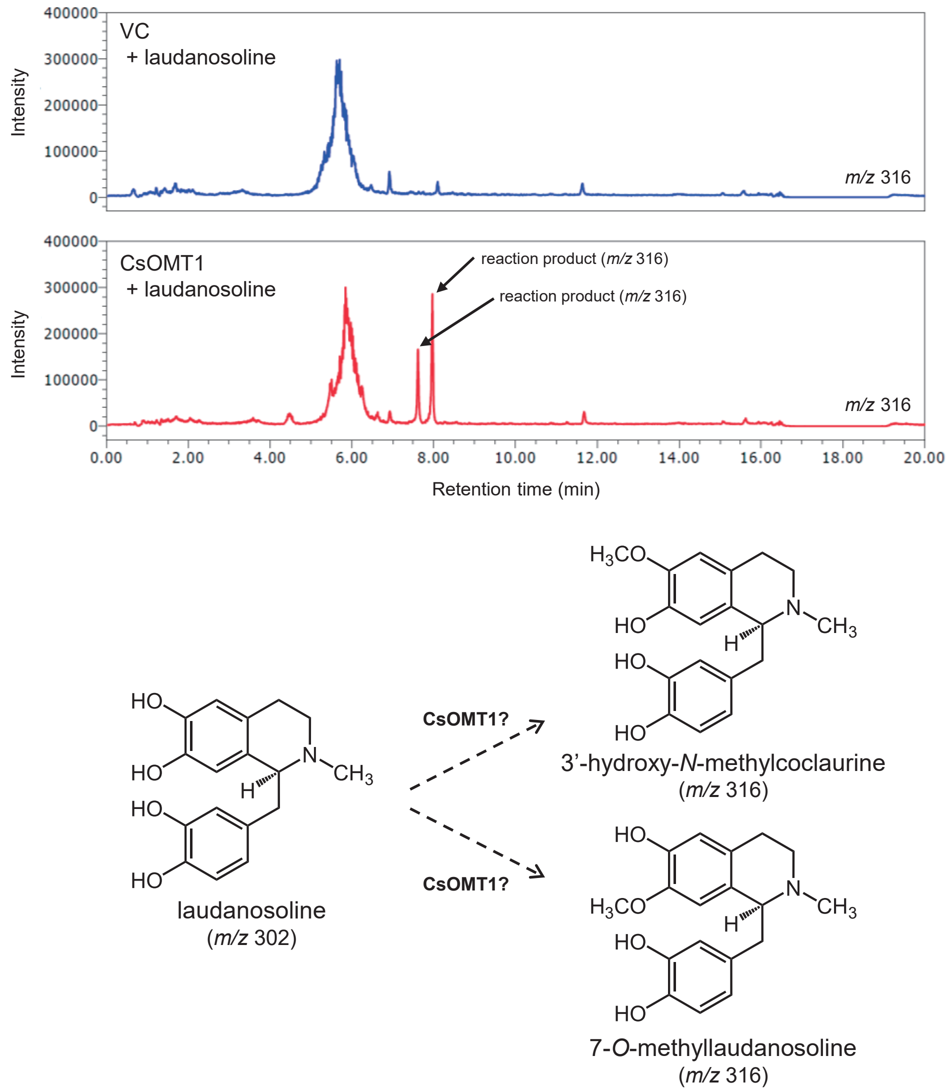

Supplementary Figure S6. Reaction products of CsOMT1 with laudanosoline.

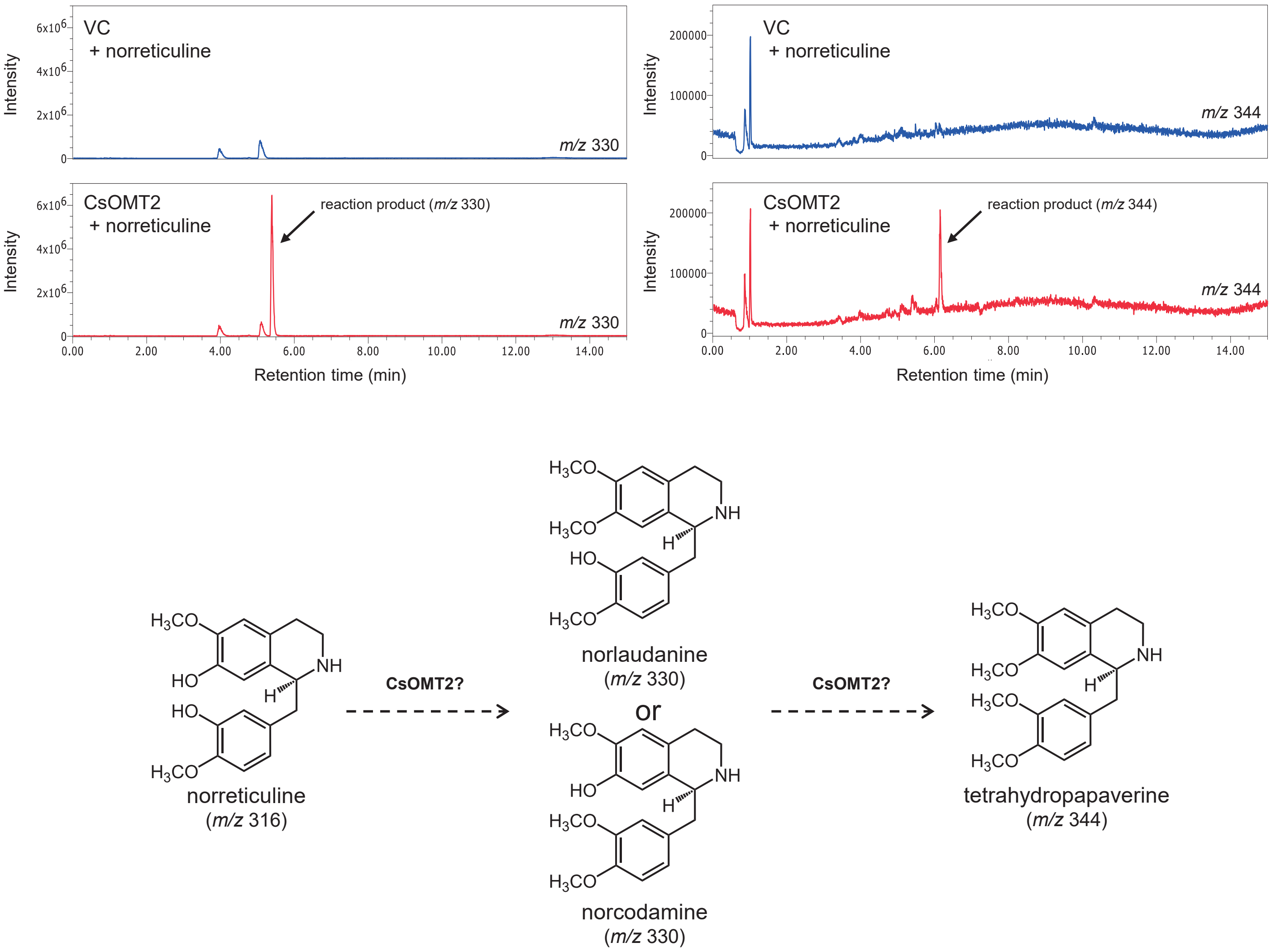

Supplementary Figure S7. Reaction products of CsOMT2 with norreticuline.

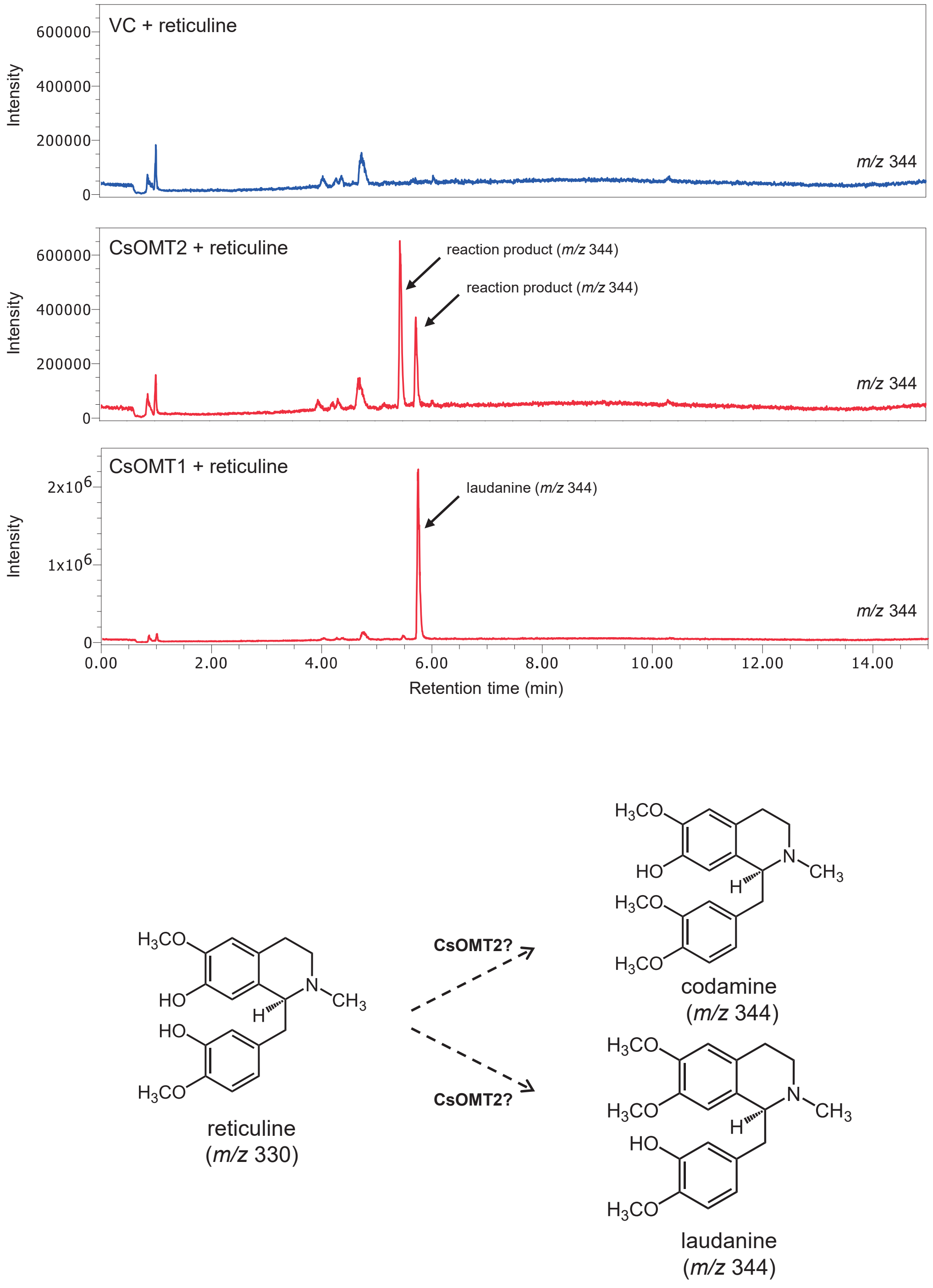

Supplementary Figure S8. Reaction products of CsOMT2 with reticuline.

Supplementary Table S1. Primer sequences used for isolation of cDNAs

Primer name	Oligonucleotide sequences (5' to 3')
CsOMT1_5'UTR_Fw	CTCTCCATTGTAGACACCAAAACTAAC
CsOMT1_3'UTR_Rv	GGAATCTCATGATCCATCAGC
CsOMT2_5'UTR_Fw	CTCTCATCTCGATCTCTTTTCTCTG
CsOMT2_3'UTR_Rv	CATACGAGGGAGCATAACCCT

Supplementary Table S2. Accession numbers of OMTs used for the phylogenetic analysis

Name	Species	Accession No.
Cj6OMT	*Coptis japonica*	Q9LEL6
Cj4'OMT	*Coptis japonica*	Q9LEL5
CjCoOMT	*Coptis japonica*	Q8H9A8
CjSMT	*Coptis japonica*	Q39522
Ec4'OMT	*Eschscholzia californica*	BAM37633
Ec6OMT	*Eschscholzia californica*	BAM37634
Ec7OMT	*Eschscholzia californica*	BAE79723
EcG3OMT	*Eschscholzia californica*	BBA20643
GsOMT1	*Gloriosa superba*	QLI49050
IpeOMT4	*Carapichea ipecacuanha*	BAI79243
AmCOMT	*Ammi majus*	AAR24095
AtOMT1	*Arabidopsis thaliana*	NP_200227
CaFOMT	*Chrysosplenium americanum*	Q42654
CbCOMT	*Clarkia breweri*	O23760
CbIEMT1	*Clarkia breweri*	O04385
PaAIMT1	*Pimpinella anisum*	B8RCD3
MdOMT1	*Malus domestica*	AKN09016
Ps4'OMT2	*Papaver somniferum*	XP_026440860
Ps6OMT	*Papaver somniferum*	XP_026389064
Ps7OMT	*Papaver somniferum*	XP_026440002
PsN7OMT	*Papaver somniferum*	XP_026431254
PsSOMT1	*Papaver somniferum*	I3V6A7
PsSOMT2	*Papaver somniferum*	NP_001392355
PsSOMT3	*Papaver somniferum*	NP_001392351
Tf6OMT	*Thalictrum flavum*	Q5C9L7
NnOMT1	*Nelumbo nucifera*	XP_010244054
NnOMT5	*Nelumbo nucifera*	XP_010276063
*[1]AcOMT1	*Aristolochia contorta*	(unregistered)
Tf4'OMT	*Thalictrum flavum*	AAU20768
TfSOMT	*Thalictrum flavum*	AAU20770
ObEOMT	*Ocimum basilicum*	Q93WU2
*[2]CyOMT2	*Corydalis yanhusuo*	(unregistered)
*[2]CyOMT5	*Corydalis yanhusuo*	(unregistered)
*[2]CyOMT6	*Corydalis yanhusuo*	(unregistered)
*[2]CyOMT7	*Corydalis yanhusuo*	(unregistered)
ObF8OMT1	*Ocimum basilicum*	S5DWK8
EgCOMT	*Eucalyptus gunnii*	P46484
Os3'FOMT	*Oryza sativa*	WBQ36416
Cr16OMT	*Catharanthus roseus*	B0EXJ8
GflOMT1	*Glaucium flavum*	AKO60152
GflOMT2	*Glaucium flavum*	AKO60153
GflOMT6	*Glaucium flavum*	AKO60157
GsOMT2	*Gloriosa superba*	QLI49054
GsOMT3	*Gloriosa superba*	QLI49055
GsOMT4	*Gloriosa superba*	QLI49057
EcG11OMT	*Eschscholzia californica*	BBA20642
NnOMT6	*Nelumbo nucifera*	XP_010241050
NnOMT7	*Nelumbo nucifera*	XP_010263004

*1 Sequence of AcOMT1 was retrieved from Cui et al. 2022

*2 Sequences of CyOMT2, CyOMT5, CyOMT6, and CyOMT7 were retrieved from Bu et al. 2022

References

Bu J, Zhang X, Li Q, Ma Y, Hu Z, Yang J, Liu X, Wang R, Jiao X, Chen T, et al. (2022) Catalytic promiscuity of *O*-methyltransferases from *Corydalis yanhusuo* leading to the structural diversity of benzylisoquinoline alkaloids. *Hortic Res* 9: uhac152

Cui X, Meng F, Pan X, Qiu X-Y, Zhang S, Li C, Lu S (2022) Chromosome-level genome assembly of *Aristolochia contorta* provides insights into the biosynthesis of benzylisoquinoline alkaloids and aristolochic acids. *Hortic Res* 9: uhac005
